# Peptide Tectonics: Encoded Structural Complementarity Dictates Programmable Self‐Assembly

**DOI:** 10.1002/advs.201802043

**Published:** 2019-04-29

**Authors:** Shaofeng Lou, Xinmou Wang, Zhilin Yu, Linqi Shi

**Affiliations:** ^1^ Key Laboratory of Functional Polymer Materials, Ministry of Education State Key Laboratory of Medicinal Chemical Biology Institute of Polymer Chemistry College of Chemistry Nankai University Weijin Road 94 Tianjin 300071 China

**Keywords:** biomaterials, conformational entropy, hierarchical nanostructures, peptides, self‐assembly, supramolecular chemistry

## Abstract

Programmable self‐assembly of peptides into well‐defined nanostructures represents one promising approach for bioinspired and biomimetic synthesis of artificial complex systems and functional materials. Despite the progress made over the past two decades in the development of strategies for precise manipulation of the self‐assembly of peptides, there is a remarkable gap between current peptide assemblies and biological systems in terms of structural complexity and functions. Here, the concept of peptide tectonics for the creation of well‐defined nanostructures predominately driven by the complementary association at the interacting interfaces of tectons is introduced. Peptide tectons are defined as peptide building blocks exhibiting structural complementarity at the interacting interfaces of commensurate domains and undergoing programmable self‐assembly into defined supramolecular structures promoted by complementary interactions. Peptide tectons are categorized based on their conformational entropy and the underlying mechanism for the programmable self‐assembly of peptide tectons is highlighted focusing on the approaches for incorporating the structural complementarity within tectons. Peptide tectonics not only provides an alternative perspective to understand the self‐assembly of peptides, but also allows for precise manipulation of peptide interactions, thus leading to artificial systems with advanced complexity and functions and paves the way toward peptide‐related functional materials resembling natural systems.

## 1 Introduction

Synthetic peptides either derived from the subunits of native proteins or created by de novo design have been utilized as building blocks for self‐assembly to construct a wide variety of complex nanostructures from the bottom up and develop functional materials.^1^ Conventionally peptide sequences consisting of amide bond–linked residues could adopt specific conformation in defined media, primarily including α‐helix, β‐sheet, and random coil. Combining the conformational adaptability of peptides with the diversity of their component residues ranging from standard and noncanonical amino acids to synthetic counterparts,^2^ one could readily create a broad library of peptides with desirable biochemical (reactivity, chirality, and toxicity) and physical (size, shape, and conformation) properties. The structural features of peptides, predominantly referring to amide bonds, allow them to spontaneously undergo self‐assembly mainly driven by noncovalent interactions, including van der Waals, hydrophobic, hydrogen bonding, electrostatic, and π–π stacking interactions, as well as metal coordination.^3^ The dynamic nature of noncovalent interactions between peptides renders resulting supramolecular structures stimulus‐responsive, self‐adaptable, or self‐healing, thus being utilized as functional materials with potential in many fields,^4^ particularly for biomaterials due to their biological origin and derived biofunctions.^5^

**Scheme 1:**
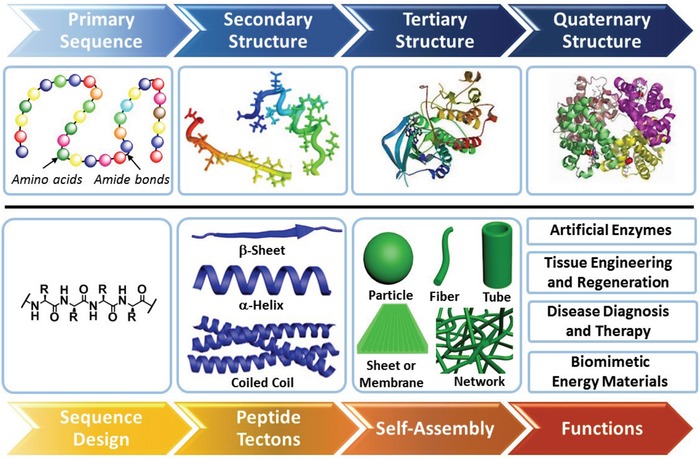
The pathway in biological systems for synthesis of sophisticated proteins and the strategy of peptide tectonics to create comparable bioinspired or biomimetic systems via rational control over the interfacial association between structural complementarity at predictable interfaces.

Despite the ubiquity of peptide interactions, there is a considerable gap between peptide self‐assembly and natural systems in terms of the toolkit for precise control over structural features, thus limiting the function of resulting materials. This gap could be primarily attributed to the difference in the self‐assembly mechanism that is associated with the structural features. In native proteins, the primary sequences of proteins are synthesized via gene transcription and translation steps and adopt specific ordered secondary structures predominantly stabilized by H‐bonds (**Scheme** 1). Subsequently, individual secondary structures interact at the structurally complementary recognizing interfaces to form the tertiary structures termed as protein subunits. Eventually, multiple subunits integrate into one entire system exhibiting dynamic global conformation and functions for essential biological processes. In contrast, peptide building blocks for self‐assembly mostly consist of primary sequences that are less ordered and lack structural complementarity. Thus, the selective association among combined peptide building blocks is poor, further preventing their programmable self‐assembly at one level comparable to native protein systems. Therefore, elucidation of a reliable sequence–structure–function relationship in peptide assemblies and resulting biomaterials remains challenging.

**Scheme 2:**
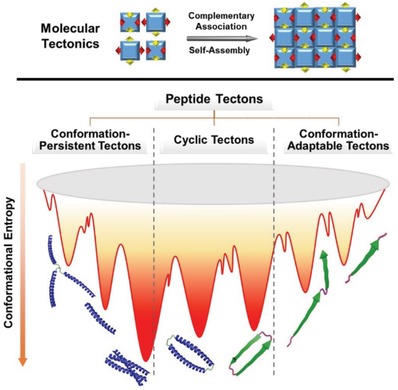
Schematic representation of (top) molecular tectonics to create 2D networks driven by complementary noncovalent interactions and (bottom) the classification of peptide tectons into three categories based on the conformational stability of incorporated domains and their conformational space, which simultaneously govern the conformational entropy of tectons. The persistence of the conformation of peptide tectons refers to the secondary structures retained in the monomeric and assembled states.

Paving the way of programmable peptide self‐assembly to nanostructures resembling native system with defined complexity and functions lies in establishing reliable strategies by which the interaction between peptide building blocks could be precisely governed at the molecular level (Scheme 1). One of promising strategies to understand the peptide association stems from molecular tectonics that was developed by J. D. Wuest and M. W. Hosseini, among others, and aims to generate molecular networks in the crystalline state based on organization of tectons capable of selective association primarily guided by hydrogen bonding, metal coordination, or host–guest inclusion (**Scheme** 2).^6^ Wuest defined molecular tectonics as *interrelationships in complex forms built from multiple subunits that retain their identity in the final structures*.^7^ On the other hand, Hosseini stated that molecular *tectons are active building units bearing recognition information and thus capable of recognizing each other*. Based on the definition of molecular tectonics and tectons, many building blocks with conformational rigidity or flexibility have been designed and utilized in creation of molecular networks. The rigid tectons exemplified by aromatics and porphyrins exhibit little conformational entropy, whereas the flexible tectons including pseudocrown ethers derived from oligo(ethylene glycol) units might undergo conformational transition during metal coordination. As the representative pioneering work in synthetic biology, Woolfson and colleagues have illustrated the role of general biomolecular tectonics in creation of synthetic biological systems based on broad natural basic units.^8^ Despite a limited number of examples for creating nanostructures^9^ or outlining the progress of the self‐assembly of coiled coils,^10^ the terminology of peptide tectonics is promising to rationalize creation of well‐defined peptide nanostructures.

To facilitate precise creation of well‐defined peptide nanostructures, we attempt to overview the peptide self‐assembly based on the concept of peptide tectonics by focusing on the intermolecular interaction arising from the complementary association between building blocks. To clarify the covering field of peptide tectonics, here we define peptide tectons as building blocks exhibiting structural complementarity at the interacting interfaces of commensurate domains to promote selective association and undergoing programmable self‐assembly following predictable organizing patterns into defined supramolecular structures (Scheme 2). In our definition, peptide tectons could be created from either single or multiple domains that adopt identical or distinct conformation, which is in contrast to the conventional strategy for design of peptide monomers focusing on specific secondary structures. Considering the fact of the potential conformational fluctuation of peptides before and after self‐assembly, both domains with a stable conformation and those undergoing a conformational transition during self‐assembly could be incorporated into tectons. The selective association at the interacting interfaces between peptide tectons can be established based on the preferential interaction arising from their structural complementarity. The most sufficient strategy for the interface match could be accomplished by rational incorporation of amino acid residues to generate the hydrophobic interface composed of nonpolar residues or the electrostatic interfaces involving positively and negatively charged residues.[qv: 3a–c] In addition, metal coordination^11^ and covalent capture^12^ are two alternative approaches to create the structural complementarity for preferential interaction at controllable interfaces. Based on the position of complementary units within peptide tectons, the selective association at the interacting interfaces could promote longitudinal, lateral, or orthogonal 2D elongation of peptide tectons into well‐defined nanostructures. Combining the enriched types of incorporated secondary structures with the precise control over organization of peptide tectons potentially facilitates creation of well‐defined nanostructures. Hence, peptide tectonics potentially paves the way toward development of peptide nanostructures resembling proteins that typically consist of different secondary structures.

Incorporation of the structural complementarity into peptide tectons at their interacting interfaces benefits from prediction of the organizing pattern of tectons. The probability to predict organizing pattern of tectons is associated with the inherent conformation of peptide domains and their conformational space. It is reasonable that both increase of the conformational stability and restriction of the conformational space of peptide domains lower the conformational entropy of peptide tectons, thus allowing for readily predicting the organizing patterns and interacting interfaces of tectons and thereby facilitating incorporation of structural complementarity at desirable interfaces. Taking the conformational stability of peptide domains and their conformational space into account, we classify peptide tectons into three categories: 1) conformation‐persistent peptide tectons; 2) conformation‐adaptable peptide tectons; and 3) cyclic peptide tectons (Scheme 2). In the cases of peptide tectons consisting of stable secondary structures, the conformation of incorporated domains is maintained before and after self‐assembly, thus leading to conformation‐persistent peptide tectons. In contrast, creation of peptide tectons from domains undergoing a conformational transition during self‐assembly results in conformation‐adaptable peptide tectons. In the former case, the retained conformation of domains allows to readily predict the organizing patterns and incorporate structural complementarity at interacting interface of tecton. However, in the latter case, the thermodynamically favorable conformation of domains within assemblies is different from that in monomeric state. Hence, the complementary units are typically incorporated into peptide tectons at the interacting interfaces of presumable stable secondary structures within assemblies. This somehow gives rise to the difficulty in design of the conformation‐adaptable peptide tectons due to the challenge in prediction of the conformational transition of domains. In addition to the aforementioned two categories, peptide tectons could be created by cyclic domains, which possess restricted conformational space and lowered conformational entropy during the self‐assembling process. Thus far, either stable or unstable domains could be cyclized and incorporated into cyclic peptide tectons. Hence, the conformational entropy of cyclic peptide tectons is dependent on the incorporated domains.

In this review, we summarize the progress of peptide tectonics in creation of well‐defined hierarchical nanostructures driven by complementary association of tectons at interacting interfaces. We outline the categories of peptide tectonics based on the conformational entropy of tectons at the monomeric level during the assembling process. We focus on the strategies by which the structural complementarity could be incorporated into peptide tectons to promote the recognizing interaction at specific interacting interfaces and guide the self‐assembly of peptide tectons. Despite the broad literature on peptide self‐assembly into nanostructures, it is worth noting that this review only covers the remarkable examples of creation of hierarchical nanostructures based on the *selective association* of peptides (beyond natural proteins and peptide conjugates) resulting from structural complementarity. Development of functional biomaterials, such as artificial cellular matrices, antimicrobial agents, and gene delivery, will be briefly discussed, indicative of the broad influence of peptide tectonics in the fields ranging from peptide nanotechnology to materials science.

## 2 Conformation‐Persistent Peptide Tectons

Structure‐persistent building blocks are broadly designed and synthesized to create well‐defined nanostructures, ranging from synthetic organic building blocks to natural proteins. In the cases of peptide tectons, the defined structural features of conformation‐persistent peptide tectons, which consist of domains adopting stable and identical conformation in both monomeric and assembled states, facilitate prediction of potential interacting interfaces among peptide tectons and rational incorporation of associating sites at positions in demand. Derived from the folding propensity of proteins, some specific peptide domains could form stable helical structures, such as α‐helices and polyproline‐type helices, in solution at the monomeric level, thus allowing for design of the conformation‐persistent peptide tectons. Conformation‐persistent peptide tectons could be created from either single or multiple ordered peptide domains. In the cases of peptide tectons consisting of single domains, in addition to single domains serving as building blocks, tectons might initially form oligomeric tectons via noncovalent interactions to serve as the subunits of nanostructures. Nevertheless, the conformational entropy of this type of tectons is almost free during self‐assembly. In contrast, despite maintenance of the conformation of incorporated secondary structures, peptide tectons consisting of flexibly linked multiple domains might exhibit change of their conformational entropy, thus leading to the challenge in precise control over the organizing patterns of tectons. It worth noting that persistence of the conformation of peptide tectons is referred to as the incorporated secondary structures within tectons, rather than the conformation of entire tectons, which might undergo conformational fluctuation dependent on the microenvironment of domains. In addition to the components of peptide tectons, the underlying driving forces for the self‐assembly of persistent peptide tectons could be divided into a variety of reliable connecting manners, including electrostatic interactions, metal coordination, and covalent linkages, among others. Within this section, we outline the self‐assembly of the conformation‐persistent peptide tectons focusing on the conformation of incorporated domains and the primary driving forces promoting self‐assembly.

### 2.1 Coiled‐Coil Tectons

Coiled coils are stable oligomers formed by multiple α‐helical strands with the *abcdefg*‐heptad repeats predominantly driven by hydrophobic interactions among the nonpolar residues sandwiched by hydrophilic faces composed of peripheral polar residues.^13^ However, the further aggregation of individual coiled‐coil bundles is prevented due to their blunt ends. Inspired by the staggering overlap of natural protein subunits, design of peptide tectons composed of coiled coils with active ends could facilitate their programmable self‐assembly into hierarchical nanostructures.^14^ Here, we summarize the self‐assembly of coiled‐coil tectons based on different connecting strategies.

#### 2.1.1 Electrostatic Interactions

**Figure 1:**
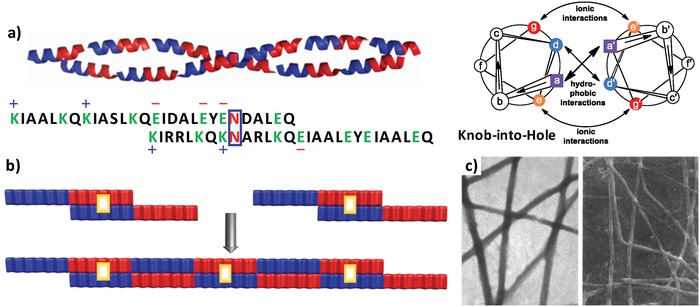
a) SAF coiled coils consisting of two linear peptide tectons with complementary charges. b) The electrostatic interactions, in combination with the H‐bonds involving the amide group of Asn residues, led to formation of staggered, parallel heterodimers with “sticky ends.” c) Nanofibers formed by SAF coiled coils. The yellow rectangles in (b) denote the Asn pairs. Reproduced with permission.^14^ Copyright 2000, American Chemical Society.

As a pioneering strategy to generate active ends for coiled coils, Woolfson and coworkers established a concept, namely, self‐assembling fiber (SAF) systems, to design coiled‐coil heterodimers decorated with complementarily charged sticky ends (**Figure** 1),^14^ inspired by the design of DNA sticky ends.^15^ The SAF systems consist of two linear complementary de novo peptides that contain four heptad repeats. While localizing Ile and Leu residues at the *a* and *d* positions of the hydrophobic core of the two peptides strengthens the “knobs‐into‐holes” interaction, incorporation of Lys and Glu residues into the two SAF peptides at the *e* and *g* positions of the N‐terminal or C‐terminal two‐heptad repeats, respectively, promotes their selective stagger between the N‐terminal and C‐terminal halves. This stagger could be enhanced by introduction of one Asn residue into the N‐terminal or C‐terminal half of the two peptides at their *a* position, due to the H‐bonds formed between the amide side chain of Asn and the coiled‐coil cores.^16^ As a result, the two peptides formed staggered and parallel heterodimers, thus further longitudinally assembling into long nanofibers. This strategy has also been utilized to create a variety of nanostructures composed of coiled coils.^17^

**Figure 2:**
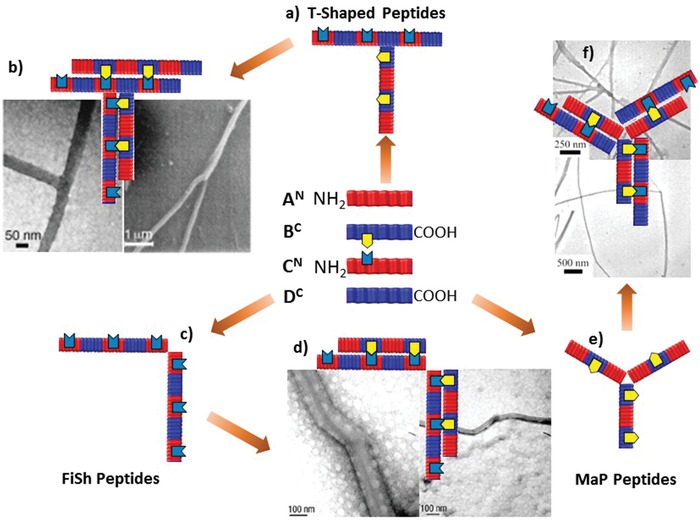
Design of a) T‐shaped, c) FiSh, and e) MaP peptide tectons starting from the SAF peptides with complementary charges. b,d,f) Using four SAF domains, i.e., A^N^, B^C^, C^N^, and D^C^ (the superscripts denote the free terminus of the domains in entire peptides), T‐shaped, FiSh, and MaP peptide tectons were designed via rational combination and coassembled with the SAF peptides into branched or networked coiled‐coil nanostructures. Yellow and red signals denote the Asn residues within the B^C^ and C^N^ half domains, respectively. a,b) Reproduced with permission.^18^ Copyright 2003, Wiley‐VCH. c,d) Reproduced with permission.^19^ Copyright 2003, Nature Publishing Group. e,f) Reproduced with permission.^20^ Copyright 2005, American Chemical Society.

Based on this concept, Woolfson and coworkers have developed peptide tectons consisting of multiple domains connected by flexible linkages, including T‐shaped,^18^ fiber‐shaping (FiSh),^19^ and matrix‐programming (MaP) peptides,^20^ to spawn branches and kinks during self‐assembling processes (**Figure** 2). The T‐shaped peptides were created by attaching the C^N^ half to the SAF‐p2 peptide via three Ala units between the C‐terminus of C^N^ half and the ε‐amino group of the central Lys residues of SAF‐p2 (Figure 2a,b).^18^ The attached C^N^ half in the T‐shaped peptides served as additional interacting sites for SAF‐p1 peptides to promote orthogonal self‐assembly into branched fibers. The percentage of branched nanostructures is associated with the equivalent of T‐shaped peptides to SAF peptides, in which a mixture of SAF peptides and T‐shaped peptide in an equimolar ratio formed 30% branched fibers. However, the branching density of individual fibers is significantly limited, due to the low compatibility of the T‐shaped peptides within the SAF systems. This was addressed by creating new peptide tectons that could be integrated into the SAF assemblies with low energy barriers, namely, the FiSh peptides (Figure 2c,d).^19^ FiSh peptides were designed by connecting two identical half domains of the standard SAF peptides in a head‐to‐head or tail‐to‐tail fashion, leading to C_2_^N^ or D_2_^C^ FiSh peptides. The termini of FiSh peptides are free within SAF peptides, thus allowing for parallel integration of the FiSh and SAF peptides. During the coassembly of FiSh and SAF peptides, FiSh peptide acted as the nucleating or elongating sites for the SAF peptides to converge and diverge the fiber growth, leading to kinked and branched nanofibers. To further increase the splitting density and create cross‐linking sites in resulting nanostructures, the Woolfson laboratory developed a strategy involving MaP peptides to produce interconnecting peptide networks (Figure 2e,f).^20^ The MaP peptides could combine the advantages of FiSh and T‐shaped peptides for generating the kinking or branching sites within fibers and retaining the branch length. Unfortunately, the fibrillogenesis in rational control over the geometry of nanofibers by the MaP peptides remains challenging, potentially due to the thermodynamically unfavorable introduction of structural defects into the crystalline peptide structures formed by SAF peptides.^21^

**Figure 3:**
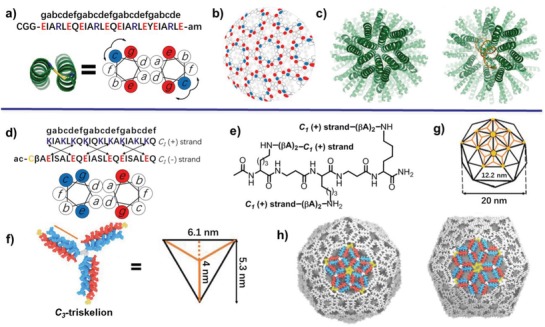
Design of virus‐like architectures. Top: a) Sequence of the trifaceted coiled‐coil tecton and its coiled‐coil subunit under helical wheel pattern with 3.5 residues per turn. The cysteine bridge is highlighted in yellow. The clockwise and anticlockwise arrows indicate intra‐ (*c*–*g*) and interhelical (*c*–*e*′) electrostatic interactions, respectively. b) Formation of virus‐like shells by assembling the trifaceted helix tectons at three facets and cross‐linking cysteine residues. c) 3D ribbon models of virus‐like shells assembled without (left) and with siRNA (right). Reproduced with permission.^22^ Copyright 2016, American Chemical Society. Bottom: d) Sequence of the positively charged C_1_(+) chain in the dendrimer C_3_‐hub and the negatively charged C_1_(−) chain aligned under the coiled‐coil heptad repeat pattern (top) and the helical wheel pattern (bottom). The cysteine unit is highlighted in yellow. e) The chemical structure of the dendrimer C_3_‐hub (C_3_(+) strand). βA refers to β‐alanine. f) Molecular model of the C_3_‐triskelion subunit. Each subunit corresponds to the altitude of an equilateral triangle. g) *T* = 4 icosahedron with four facets in each triangular face occupied by one triskelion. h) Model of a *T* = 4 capsid assembled from the C_3_‐triskelion, in which one of the fivefold (left) or sixfold (right) axes is highlighted. Reproduced under the terms of the Creative Commons 4.0 license.^23^ Copyright 2017, Nature Publishing Group.

Combining the complementary electrostatic interactions with topology control allows for design of complex peptide tectons. For instance, Ryadnov and coworkers have designed and synthesized a trifaceted coiled‐coil tecton exhibiting one hydrophobic interface and two polar facets to create virus‐like shells (**Figure** 3, top).^22^ Each helix tecton associates with other three neighbor counterparts via hydrophobic and electrostatic interactions in three directions and serves as a branching cell within a continuous network (Figure 3b). Due to the requisite of the simultaneous association at all three facets of the tecton to form the network, no sticky ends can be retained within the resulting network, thus facilitating the closure of the curved networks into shells. Cross‐linking the N‐terminal cysteine units either between the same or neighbored coiled‐coil dimers into disulfide bonds on the network sheets allows for further stabilization of the resulting shells, and thereby converting the anisotropic assemblies to C_3_‐symmertic networks resembling 3D virus architectures (Figure 3c). Comparing to conventional cationic gene vehicles, the resulting virus‐like shell displayed extraordinary capability in encapsulating and transferring genetic materials into human cells and mediating gene silencing and transgene expression, demonstrating great potential in gene therapy.

The Ryadnov group has further developed the virus‐like design toward creation of antimicrobial peptide capsids via self‐assembly of a rigid C_3_‐triskelion consisting of a positively charged dendrimer C_3_(+)‐hub containing three copies of C_1_(+) chains and a linear negatively charged C_1_(−) chain in a molar ratio of 1:3 (Figure 3, bottom).^23^ The rigid C_3_‐triskelion subunits were stabilized by formation of three heterodimers promoted by the hydrophobic interactions between isoleucine/leucine residues at *a*/*d* sites as well as the electrostatic interactions between the complementary cationic lysines and anionic glutamates at *g*/*e*′ sites (Figure 3d–f). Capping the negatively charged C_1_(−) chain with a cysteine unit allows for promotion of the self‐assembly of the C_3_‐triskelion subunits into predominant capsid architectures with a triangulation number *T* = 4, driven by the hydrophobic clustering of cysteine units (Figure 3g,h). Under physiological conditions, the capsids disassembled into C_3_(+) triskelions and C_1_(−) chains, serving as the antimicrobial or antagonist agents, respectively, due to the unfolding behavior of the C_1_(−) chains. This disassembling behavior of C_3_‐capsids allows C_3_(+) triskelions to selectively associate with bacterial membranes and further promote membrane lysis, thus resulting in the remarkable antibacterial activity of C_3_‐capsids.

#### 2.1.2 Metal Coordination

**Figure 4:**
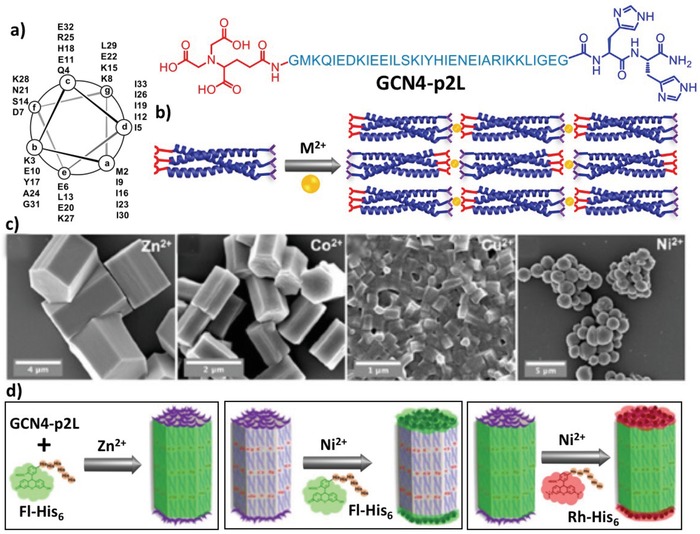
Coiled‐coil peptides GCN4‐p2L functionalized with NTA and His_2_ ligands at termini. a) Helical wheel representation and sequence of the GCN4‐p2L peptide. b) Schematic illustration of the metal coordination–triggered head‐to‐tail assembly of the GCN4‐p2L trimeric coiled coil. c) Scanning electron microscopy (SEM) images of the nanostructures formed by peptide GCN4‐p2L and divalent metal ions in a molar ratio of 1:0.4. d) Graphical representation of the spatial capture of cargoes within the nanostructures. Reproduced with permission.^26^ Copyright 2016, American Chemical Society.

Due to the directionality and reversibility of metal–ligand coordination, rational incorporation of metal ligands into peptide backbones is a versatile means to specify the association among peptide building blocks. Natural amino acids including histidine (His), aspartic acid (Asp), methionine (Met), glutamic acid (Glu), and cysteine (Cys), and synthetic ligands such as terpyridine (Tpy), bipyridine (Bpy), catechol, and nitrilotriacetic acid (NTA) have been broadly utilized as metal ligands to promote metal coordination.^24^ In the case of self‐assembly of coiled coils, the pioneering work from the Ghadiri laboratory reported one amphiphilic peptide functionalized with a 2,2′‐Bpy moiety at the N‐terminus (Bpy‐GELAEKLEQALQKLA‐NH_2_).^25^ Upon addition of Ni^2+^ or Co^2+^ ions, coordination of metal ion with bipyridyl units in a 1:3 molar ratio induced formation of stable triple helices as evident by the absorption characteristic of tri‐bipyridyl–metal complexes. This strategy was applied to design coiled‐coil tectons that undergo predictable self‐assembly into hierarchical supramolecular structures. Chmielewski and coworkers have designed and synthesized a helical peptide derived from GCN4 peptides and functionalized with one NTA and two His residues at its N‐ and C‐terminus, respectively, leading to GCN‐2pL (**Figure** 4).^26^ Thus, the negatively charged NTA and positively charged dihistine (His_2_) ligands were neighbored to oppositely charged heptad repeats to mediate the dipole of peptides. Peptide GCN‐2pL initially formed trimeric coiled coils that further underwent the programmable self‐assembly in a linear head‐to‐tail array caused by the longitudinal coordination between divalent metal ions and terminal ligands. Varying the type of metal ions or the molar ratio between metal ions and peptides led to formation of microscale hexagonal 3D crystal or nano‐/microspheres (Figure 4c). X‐ray diffraction experiments elucidated that within the hexagonal crystals different strands associated via hydrogen‐bonding interactions were orientated in an antiparallel direction due to the electrostatic interactions between surface‐exposed charged moieties. The head‐to‐tail organization of the coiled coils resulted in some free ligands at the ends of nanostructures, which can be accessed by additional metal ions to spatially direct inclusion of cargoes in the crystals dependent on the kinetics of metal coordination involving different divalent metal ions (Figure 4d).

**Figure 5:**
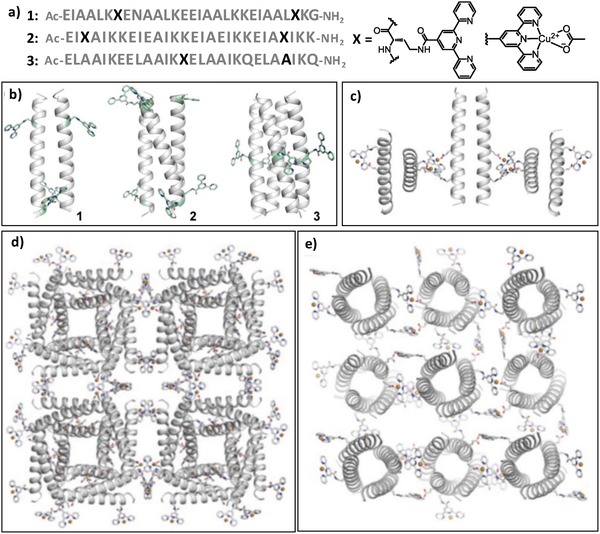
a) Sequences of peptides **1**, **2**, and **3**, chemical structure of the non‐natural residue X containing Tpy unit, and the coordinating model of the Tpy ligand with Cu^2+^ involving a Glu residue. b) Schematic representation of the dimeric, trimeric, and tetrameric coiled coils formed by peptides **1**, **2**, and **3**, respectively. Crystal structures: c) 1D coordination polymer formed by laterally organized peptide **1**, d) extended framework formed by peptide **2**, and e) 2D net formed by peptide **3** based on the Tpy–Cu^2+^–Glu linkages. Reproduced with permission.^27^ Copyright 2017, American Chemical Society.

In addition, Horne and coworkers have incorporated Tpy unit into α‐helices to create multidimensional peptide structures based on the lateral metal coordination (**Figure** 5).^27^ The localization and number of Tpy units have been optimized to tune the organizing patterns of coiled coils, leading to peptides **1** and **2** with two Tpy units at their internal or nearly terminal position, and peptide **3** with one Tpy unit at its central position, in which peptides **1**, **2**, and **3** coiled into dimers, trimers, and tetramers, respectively. Coordination of divalent metal ions with the Tpy unit and a surrounding Glu residue led to Tpy–M–Glu complexes that directed the self‐assembly of formed coiled coils. In the case of dimeric coiled coil **1**, two of the four terpyridine units guided its lateral assembly, whereas the other two governed the formation of a large lattice. The six Tpy units positioned near the termini of the trimeric coiled coil **2** facilitated the formation of an extended 3D framework. Coordinating Cu^2+^ ions with the four Tpy moieties at the center of tetrameric coiled coil **3** as well as the surrounding Glu residues yielded the Tpy–M–Glu complexes that induced formation of tetragonal units within the resulting 2D crystalline structures. This work demonstrated that altering the ligand position within peptide tectons renders the crystalline units in peptide assembled architectures different.

#### 2.1.3 Covalent Capture

**Figure 6:**
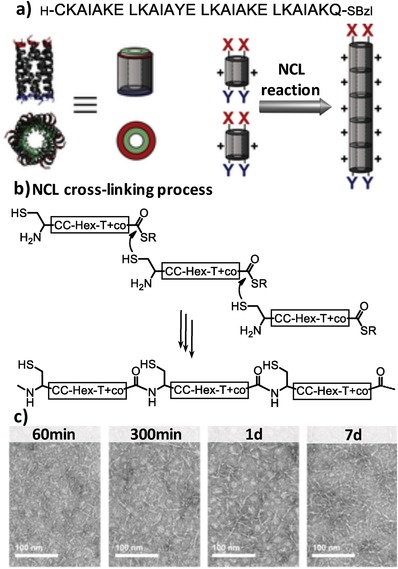
a) Peptide sequence of the coiled coils terminated with thiol and thioester groups and schematic representation of covalently linking αHBs leading to formation of long nanotubes. b) The oligomerization of αHBs induced by NCL reactions. c) Transmission electron microscopy (TEM) images of resulting nanotubes stabilized by the NCL reaction, as evident from increase of the length of nanofibers at different times. Reproduced under the terms of the Creative Commons license.^28^ Copyright 2015, Wiley‐VCH.

Natural chemical ligand (NCL) reactions have also been employed to create covalently linked coiled coils involving terminal cysteine and thioester residues (**Figure** 6).^28^ In general, functionalizing α‐helical barrels (αHBs)^29^ with lysine and glutamic acid residues at the termini of αHBs facilitated the longitudinal and lateral growth of the αHBs caused by the electrostatic and hydrophobic interactions,^30^ respectively, thereby rendering bundled peptide nanotubes. The lateral aggregation of the nanotubes can be prevented by incorporating an additional lysine unit in the central position of the αHBs, leading to dispersive nanotubes at low pH resulting from the electrostatic repulsion. Based on these established systems, Woolfson and coworkers modified the αHBs within the well‐dispersed nanotubes with N‐terminal cysteine and C‐terminal thioester moieties, thus allowing for covalent NCL connection of the αHB monomers into stable αHB polymers up to 100 nm in length.^28^ The covalently linked αHB nanotubes enable efficient capture of hydrophobic guest molecules into the hollow cavity, due to the high binding affinity associated with the structural stability.

### 2.2 Collagen‐Mimetic Peptide Tectons

Collagen proteins, known as the most abundant protein family in body and the predominant component of connective tissues, are formed by three identical or distinct polypeptide strands, which adopt a left‐handed type‐II polyproline helix conformation, wrapping around one another in a right‐handed twisted manner. Inspired by collagen triple helices, small collagen‐mimetic peptides (CMPs) that are able to form stable hetero‐ or homo‐triple helices in solution have been developed for the study of the inherent structural features of collagen proteins as well as creation of hierarchical nanostructures. The stability of formed triple helices allows for design of conformation‐persistent peptide tectons from CMPs. The self‐assembly of CMP tectons could be promoted through various connecting methods. Note that, to be consistent with the definition of conformation‐persistent structures, this section covers only the examples in which the CMP tectons undergo self‐assembly into hierarchical nanostructures. The references reporting the formation of stable CMP triple helices will not be discussed here.

#### 2.2.1 Electrostatic Interactions

**Figure 7:**
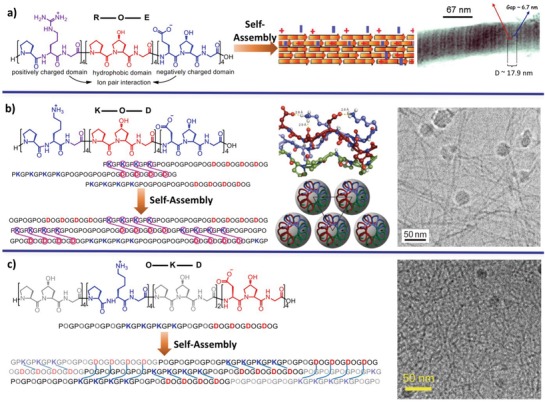
Linear CMP tectons consisting of the domains with complementary charges. Chemical structures for the CMPs containing a) PRG and EOG (R–O–E model) or b) PKG and DOG domains (K–O–D model) linked by a neutral POG domain, as well as c) PKG and DOG domains linked by two neutral POG domains (O–K–D model). The representative stacking fashion of individual CMPs stabilized by the salt‐bridge interactions, and the corresponding morphology of the resulting nanostructures investigated by TEM are shown. a) Reproduced with permission.^33^ Copyright 2007, American Chemical Society. b) Reproduced with permission.^34^ Copyright 2011, Nature Publishing Group. c) Reproduced with permission.^35^ Copyright 2011, Nature Publishing Group.

Analogous to the self‐assembly of coiled coils, creation of sticky ends for CMP triple helices is critical for promotion of their self‐assembly. The pioneering work was reported by the Raines laboratory by tethering the fragment strands via disulfide bonds to arrange the three strands in a staggered array, thus allowing for formation of intermolecular triple helices and promotion of self‐assembly of CMPs into nanofibrils.^31^ Alternative to the covalent tethering strategy, complementary electrostatic interaction has been employed to direct the self‐assembly of CMP triple helices based on the stagger of complementary domains.^32^ Conticello and Chaikof reported the self‐assembly of CMP tectons containing two oppositely charged domains (Glu and Arg residues as negative and positive charges) linked by a neutral core into D‐periodic microfibers predominately driven by electrostatic and H‐bond interactions (**Figure** 7a).^33^ Hartgerink and coworkers tuned the interchain distance between the charged residues within the CMP triple helices by replacing Glu and Arg with Asp and Lys residues, respectively, resulting in formation of multihierarchical self‐assembly into nanofibers (Figure 7b).^34^ Through the mathematics of tessellations, Raines and coworkers have optimized the relative sequence and length of the Asp‐ or Lys‐containing and neutral domains in CMP molecules to maximize the salt‐bridged interactions (Figure 7c).^35^ This design created triple helices with uniform sticky ends, leading to formation of symmetric nanofibers in micrometer scale. Alternative to natural amino acids, changing the positively charged Arg residue to 4‐amino‐proline unit, while retaining Glu as the complementary residue, gave rise to formation of well‐defined nanosheets composed of laterally aligned CMPs.^36^ The thickness of the nanosheets could be tuned by changing the length of the hydrophobic domain within the CMPs. In addition, the electrostatic interaction has also been utilized to guide the self‐assembly of asymmetric linear CMP tectons. Conticello and coworkers reported a pair of CMPs terminated with a positively charged (Pro–Arg–Gly)_3_ or negatively charged (Glu–Hyp–Gly)_3_ triad domain, respectively.^37^ While individual charged CMP tectons assembled into monolayer nanosheets, addition of 2 equiv. of negatively charged CMPs into the preformed positively charged nanosheets led to formation of defined triple‐layer nanosheets, in which the positively charged monolayer was sandwiched with two negatively charged layers.

#### 2.2.2 Metal Coordination

**Figure 8:**
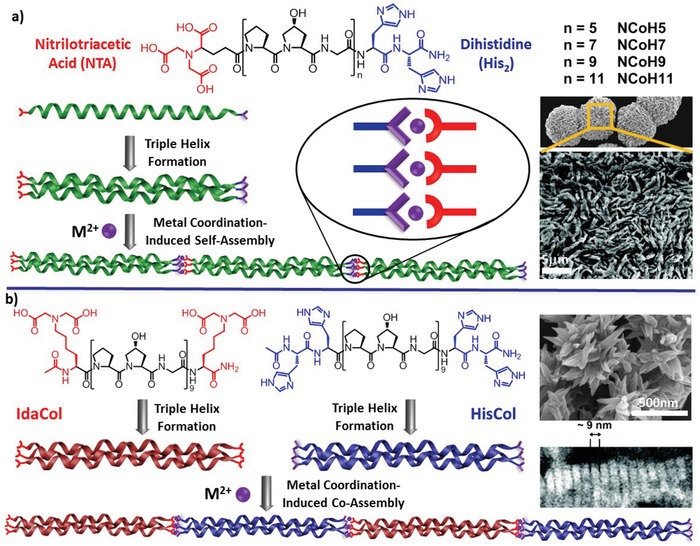
a) Self‐assembly of CMPs functionalized with NTA and His_2_ ligands at termini (**NCoH**) promoted by metal coordination into microflorettes formed by **NCoH** and Zn^2+^ ion in a molar ratio of 1:0.4. Reproduced with permission.^40^ Copyright 2009, American Chemical Society. b) Self‐assembly of a pair of symmetric CMPs functionalized with Ida (**IdaCol**) and His_2_ (**HisCol**) ligands induced by metal coordination into petal‐like nanostructures in the presence of 2 equiv. of Zn^2+^. Reproduced with permission.^42^ Copyright 2011, American Chemical Society.

An alternative sufficient strategy for creating sticky ends for the self‐assembly of stable CMP triple helices is based on metal coordination. In discrete oligomers, the CMP triple helices can be stabilized via the metal coordination involving the incorporated ligands such as Bpy or catechol units at only one terminus.^38^ Thus, the longitudinal self‐assembly of CMP peptides can be realized by incorporating metal ligands at their both termini, as illustrated by the systematic work from the Chmielewski laboratory (**Figure** 8).^39^ Initially, they designed and synthesized a CMP peptide consisting of nine repeating units of Pro–Hyp–Gly tripeptide and functionalized with two distinct ligands, i.e., NTA and His_2_ ligands, at each terminus, termed as peptide **NCoH** (Figure 8a).^40^ While the length of the amino acid sequences endows the thermal stability to the triple helices formed by peptide **NCoH** at room temperature, localization of His_2_ or NTA ligands at the termini of peptide **NCoH** sustains the longitudinal assembly of the resulting triple helices based on coordination between divalent metal ions and the combined His_2_/NTA ligands. To gain insight into the underlying mechanism of the self‐assembly of CMP peptides induced by metal coordination, Chmielewski and coworkers subsequently designed and synthesized a series of peptides **NCoH** with various number of Pro–Hyp–Gly repeating units (Figure 8a). They found that formation of supramolecular structures induced by metal coordination was intimately associated with the length of peptide **NCoH**. While addition of metal ion to short peptide **NCoH** that forms unstable triple helices did not lead to supramolecular structures, coordination of metal ions with long peptide **NCoH** that forms stable triple helices indeed resulted in microsaddle structures. This finding demonstrated that stable triple helices essentially maintain the combination of His_2_ and NTA ligands to promote metal coordination analogous to the His_2_/NTA‐lag systems, thus allowing the longitudinal self‐assembly of CMP peptides into supramolecular structures.^41^

To probe the internal organizing pattern of CMP triple helices, the Chmielewski group designed and synthesized a pair of CMP peptides with nine Pro–Hyp–Gly tripeptide units symmetrically terminated with iminodiacetic acid (Ida) or His_2_ ligands, leading to peptides **IdaCol** and **HisCol**, respectively (Figure 8b).^42^ Independent formation of stable triple helices for the two peptides led to the cluster of Ida or His ligands at the termini of **IdaCol** and **HisCol** triple helices, respectively. Mixing the preformed triple helices allows for coordination between the complementary ligands and metal ions, thus promoting longitudinal self‐assembly of triple helices. In contrast to no aggregation observed for each peptide alone in the presence of metal ions, addition of metal ions into the equimolar mixture of peptides **IdaCol** and **HisCol** resulted in increase of the solution turbidity and formation of petal‐like structures. Mixing metal ions with hetero‐triple **IdaCol**/**HisCol** helices obtained by annealing the mixture of **IdaCol** and **HisCol** triple helices did not cause formation of well‐defined nanostructures, suggesting the longitudinally alternating coassembly of segregated **IdaCol** and **HisCol** triple helices into petal‐like structures (Figure 8b).

**Figure 9:**
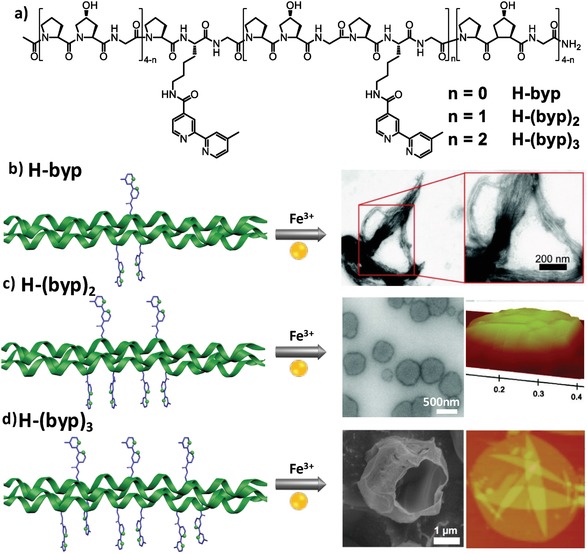
a) Chemical structures of collagen‐mimetic peptides containing one, two, or three H‐Byp at the internal tripeptide repeats, leading to CMP **H‐byp**, **H‐(byp)_2_**, or **H‐(byp)_3_**, respectively. Upon addition of Fe^3+^ ion (1 equiv. to peptides), the CMPs assembled into b) branched fibers, c) round disk‐like structures, and d) hollow spheres based on the metal coordination, in addition to the π–π stacking interactions among aromatic ligands. b) Reproduced with permission.^43^ Copyright 2008, American Chemical Society. c) Reproduced with permission.^43^ Copyright 2010, American Chemical Society. d) Reproduced with permission.^43^ Copyright 2013, American Chemical Society.

In addition to incorporation of ligands to CMPs at their termini, metal ligands have been attached to the internal tripeptide units of CMP peptides to control the lateral interaction. The Chmielewski laboratory proposed a radial assembly mechanism of CMP peptides by replacing the hydroxyproline residue with one bipyridyl‐modified lysine moiety (H‐Byp) at the internal tripeptide repeats (**Figure** 9).^43^ The number of H‐Bpy units was varied from one, two, to three, leading to peptides **H‐byp**, **H‐(byp)_2_**, and **H‐(byp)_3_**, respectively. These hanged bipyridyl units not only allowed for interstrand π–π stacking interactions, but also allowed for the bidentate coordination of bipyridine to octahedral Fe^2+^ ions, thus promoting the radial assembly of CMPs. In principle, increasing the number of aromatic ligand units strengthened the π–π stacking interactions between ligands from adjacent triple helices. Hence, in the absence of metal ions, while peptides **H‐byp** and **H‐(byp)_2_** did not or only partially aggregate,[qv: 43a,b] peptide **H‐(byp)_3_** assembled into curved disks.[qv: 43c] Upon addition of Fe^2+^ ions, peptides **H‐byp** and **H‐(byp)_2_** formed branched fibers and round disk‐like structures, respectively.[qv: 43a,b] The difference of the morphology of the metal–peptide assemblies was attributed to the flexibility between the ligands and the collagen triple helices. While the flexibly appended bipyridyl ligands in peptide **H‐byp** allow triple helices for overhang and linear growth into bundled fibers, the rigidly tethered **H‐(byp)_2_** triple helices based on the double coordinating events preferred a radial growth into disk‐like structures. In the case of peptide **H‐(byp)_3_**, addition of Fe^2+^ ions in the annealed solution of peptide **H‐(byp)_3_** led to the growth from curved disks to hollow spheres.[qv: 43c]

## 3 Conformation‐Adaptable Peptide Tectons

Comparing to stable secondary structures, design of peptide tectons from domains undergoing a conformational transition during self‐assembly increases the challenges in prediction of the organizing patterns and interacting interfaces, thus leading to difficulty in control over the programmable self‐assembly of tectons. However, over the past two decades, both understanding of the underlying kinetics and thermodynamics of peptide self‐assembly and developing the computational algorithms for theoretical simulation of the self‐assembly of peptides facilitate design of conformation‐adaptable peptide tectons. Despite the unfavorable entropic penalty resulting from the conformational transition for peptide domains during self‐assembly, the enthalpic contribution associated with the selective association arising from the structural complementarity at interacting interfaces of domains might drive the self‐assembly of peptide tectons into well‐defined nanostructures. This section covers the self‐assembly of peptide tectons consisting of domains undergoing a conformational transition from monomeric to assembled state.

### 3.1 β‐Sheet Peptide Tectons

Due to the intrinsic noncovalent interactions required for stabilizing β‐sheet secondary structures, β‐sheet domains typically adopt random coil at the monomeric state and undergo the conformational transition from random coil to β‐sheets during self‐assembly. Thus far, the comprehensive study and understanding of the structural features of β‐sheet peptides allow for design and creation of tectons from β‐sheet domains. Many reliable approaches have been utilized to promote the complementary association among β‐sheet domains, as represented by hydrophobic interactions among alternating hydrophilic and hydrophobic peptides^44^ as well as metal coordination between peptides containing ligands.[qv: 11a,b] Here, we only discuss some recent examples of the self‐assembly of peptide tectons containing multiple β‐sheet domains promoted by selective association among domains.

**Figure 10:**
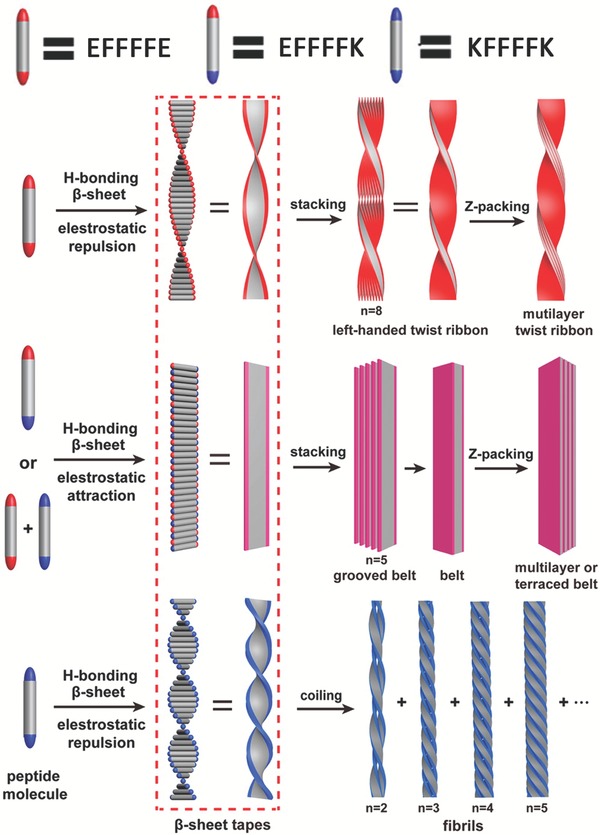
Schematic representation of the proposed mechanism of formation of twisted or flat nanostructures based on the electrostatic repulsion or attraction among peptides EF4E, EF4K, EF4E/KF4K equimolar mixture, and KF4K. Reproduced with permission.^3^ Copyright 2016, American Chemical Society.

Multidomain peptides that contain an amphiphilic‐core domain and two terminal charged domains have been conceived and developed by the Hartgerink laboratory.^45^ The amphiphilic‐core domain typically was composed of alternating hydrophobic and hydrophilic residues, thus promoting β‐sheet H‐bonds.^46^ Balancing the electrostatic repulsion among the charged terminal domains with the hydrophobic and H‐bonding interactions among the amphiphilic core allowed for formation of MDP nanofibers^47^ and thereby hydrogelation, leading to broad applications in biomedical engineering.^48^ In addition, Cui and coworkers have designed a series of short peptides consisting of a tetra‐Phe domain terminated with charged groups to precisely elucidate the effect of electrostatic interactions on control over the lamination and untwisting of β‐sheets (**Figure** 10).[qv: 3d] They found that the electrostatic repulsion between terminal charges led to highly twisted fibrils or ribbons, while the electrostatic attraction resulted in formation of flat belt‐like nanostructures.

**Figure 11:**
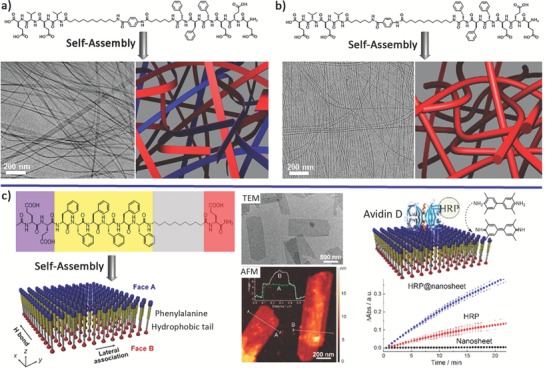
Linear asymmetric β‐sheet peptide tectons and their self‐assembly based on selective association among peptide domains. Top: Schematic representation of the self‐assembly of dual‐domain peptides into a) nanoribbons or b) nanofibers, respectively, dependent on the relative position of the alkyl tails within the isomeric linkages and the peptide domains. Reproduced with permission.^49^ Copyright 2016, American Chemical Society. Bottom: c) Self‐assembly of the linear peptides containing a hexaphenylalanine domain and an undecyl tail into Janus nanosheets, which was characterized by TEM and atomic force microscopy (AFM). The Janus nanosheets can be functionalized by biotin groups at one face to selectively capture avidin enzymes for catalyzing oxidation reactions. Reproduced under the terms of the Creative Commons license.^51^ Copyright 2017, American Chemical Society.

Multiple different β‐sheet domains can be simultaneously incorporated into linear peptide tectons to promote the selective association of domains, thus guiding the programmable self‐assembly of peptide tectons into well‐defined nanostructures. Stupp and coworkers have designed a pair of linear peptide tectons containing two isolated β‐sheet domains linked by constitutionally isomeric linkages (**Figure** 11a,b).^49^ While the two incorporated β‐sheet domains, i.e., (VE)_2_ and F_3_E_3_, preferred to form nanoribbons,^50^ the isomeric linkages were composed of a phenyl group terminated with hexyl and dodecyl tails on opposite sites. Changing the relative position between the alkyl tails and the peptide domains differentiated the strengths of noncovalent interactions, thus giving rise to formation of nanoribbons and nanofibers for strongly and weakly stacked monomers, respectively. Within the nanoribbons, preferences on self‐association of the two β‐sheet domains resulted in asymmetry of the ribbons with different peptide domains on each face. The asymmetry of the nanoribbons has been demonstrated by selectively capturing gold nanoparticles on one face when functionalizing cysteine units only at the terminus of one domain. The selective association of the domains with linear peptide tectons has been exploited by the Stevens laboratory involving one peptide amphiphile consisting of a hexaphenylalanine domain and an alkyl tail (Figure 11c).^51^ The self‐sorting between peptide β‐strands and alkyl tails led to formation of Janus 2D nanostructures with single‐layer thickness. The resulting Janus bilayer structures served as the scaffolds for selective capture of enzymes on one face for catalyzing oxidation reactions (Figure 11c).

**Figure 12:**
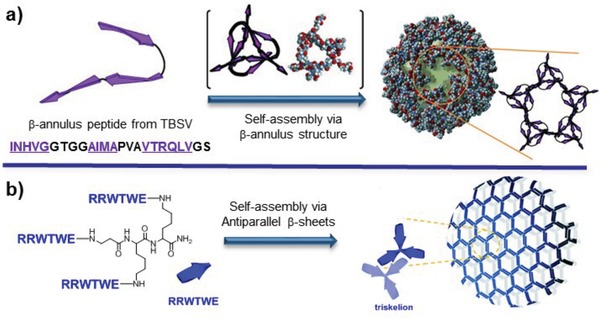
a) Self‐assembly of a β‐annulus peptide tailored from TBSV capsid into virus‐like nanocapsules. Reproduced with permission.^52^ Copyright 2010, Wiley‐VCH. b) Self‐assembly of a triskel capsid conjugate into virus‐like capsules with antimicrobial activity. Reproduced under the terms of the Creative Commons 3.0 license.^53^ Copyright 2016, Royal Society of Chemistry.

In the cases of β‐sheet tectons consisting of multidomains, Matsuura et al. have designed and synthesized a 24‐mer β‐annulus peptide (INHVGGTGGAIMAPVAVTRQLVGS) that is the Ile69–Ser92 fragment of tomato bushy stunt virus (TBSV) capsid (**Figure** 12a).^52^ The β‐annulus peptide contains three domains, i.e., INHVG, AIMA, and VTRQLV, with the propensity to form β‐sheets. Due to the sticky role of the VTRQLV domain, the β‐annulus peptide assembled into virus‐sized nanocapsules. This peptide is a minimized design of synthetic viral capsids, thus providing the opportunity for creation of artificial virus‐like nanocapsules with great potential ranging from gene or protein delivery to vaccines. Alternative to the linear topology, Ryadnov and coworkers have designed and synthesized a branching capzip tecton containing three RRWTWE chains with a trilateral symmetry reminiscent of native cage‐like subunits (Figure 12b).^53^ Starting from the core sequence RRWQWR attributing to the antimicrobial activity of lactoferrin, the authors replaced the glutamine by a threonine residue inspired by the characteristic β‐sheet core motif WTW of tryptophan zippers, and also changed the C‐terminal arginine with glutamate residue, leading to a complementary sequence RRWTWE. The resulting capzip subunits assembled into morphologically uniform hollow capsules driven by the intramolecular hydrogen bonds in the core of each capsid (β‐turn) and intermolecular hydrogen bonds between the arms of neighbored conjugates (β‐sheet). The resulting virus‐like capsules enable to serve as a vehicle to deliver gene and also exhibit antimicrobial activity, thus demonstrating a structural platform for engineering biologically differential nanomaterials.

### 3.2 β‐Hairpin Peptide Tectons

Benefitting from the confined orientation of β‐sheet strands, β‐hairpin peptides are one of ideal starting motifs to design peptide tectons to create organized nanostructures.^54^ Conventionally, two β‐sheet strands within β‐hairpins are oriented in an antiparallel direction,^55^ which allows one to encode complementary interacting sites in the two antiparallel aligned chains. While the intramolecular interactions that are formed between the inward associating sites could stabilize the folding conformation, the association between the outward interacting sites within two strands dictates lateral interacting interfaces among distinct peptide tectons, thus promoting their programmable self‐assembly into well‐defined nanostructures.

**Figure 13:**
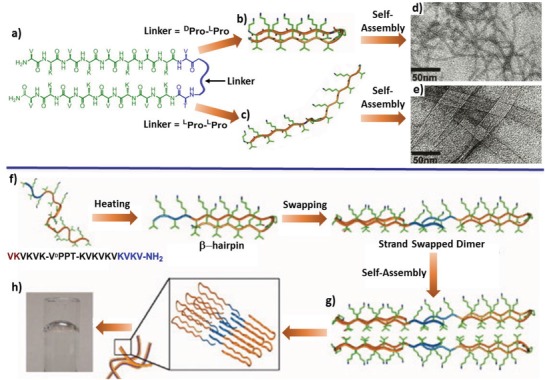
Peptide tectons derived from β‐hairpin peptides consisting of alternating valine and lysine residues flanking a –VPPT– loop. Top: a) Effect of the turn loop on peptide conformation and the morphology of resulting nanostructures, b,d) where –^D^Pro–^L^Pro– in the central turn allows the peptides to adopt a β‐hairpin conformation and self‐assemble into nanofibers, c,e) whereas peptides with –^L^Pro–^L^Pro– in the loop adopt an extended conformation and assemble into nanoribbons. d,e) TEM images of the resulting nanofibers and nanoribbons. b,d) Reproduced with permission.^56^ Copyright 2002, American Chemical Society. c,e): Reproduced with permission.^59^ Copyright 2005, American Chemical Society. Bottom: β‐Hairpins containing an exchangeable domain appended to β‐hairpin motifs. f) Sequence of the β‐hairpin and schematic representation of g) their dimerization via swapping between the exchangeable strands and h) formed hydrogels composed of nanofibrils. Reproduced with permission.^60^ Copyright 2008, American Chemical Society.

As the representative work, Schneider, Pochan, and coworkers^56^ designed β‐hairpin peptides consisting of alternating valine and lysine residues that exhibit high propensity to form β‐sheet H‐bonds flanking a central turn sequence (**Figure** 13). In their initial study, the β‐hairpin peptide contained two strands with an identical chain length linked by a –valine–^D^proline–proline–threonine– (–V^D^PPT–) loop,^57^ thus leading to a type‐II′β‐hairpin (Figure 13b). When dissolved in acidic aqueous solution, individual peptides were in an ensemble of random coil conformation due to the repulsive electrostatic interactions between the positively charged lysine residues. Upon increasing the solution pH or the ionic strength, the peptides adopted a folding conformation with valine residues toward the outside face and lysine residues toward the inside face, as a result of the release of the lysine‐based positive charges. In the folded state, the peptide underwent both lateral and facial self‐assembly driven by the H‐bonds formed between distinct hairpins and the hydrophobic association of the valine‐rich faces, respectively, leading to robust hydrogels consisting of nanofibers. The folding conformation of the peptide was also temperature‐sensitive, rendering the hydrogelation of the peptides both pH‐ and temperature‐responsive.^58^ When the central loop was changed to –VPPT–, the nature of the conformation of –^L^Pro–^L^Pro– dipeptide and the valine residue preceding the first proline gave rise to a *trans*‐propyl configuration of the turn, eventually forcing the peptide adopting an extended β‐strand conformation (Figure 13c).^59^ The extended peptides assembled into polar β‐sheets with distinct hydrophobic (valine) and hydrophilic (lysine) faces, which further laterally stacked into filaments via the collapse of hydrophobic faces. Alternative to the β‐hairpins with two strands possessing an identical chain length, the Schneider and Pochan groups designed β‐hairpins consisting of two domains with distinct lengths, in which an exchangeable domain was appended to the β‐hairpin motif (Figure 13f),^56^, ^60^ inspired by the swapped structures in native proteins.^61^ In folded states, swapping between the exchangeable strands from two distinct peptides led to a strand‐swapped dimer, which further assembled into twisted or untwisted fibrils associated with the length of the exchangeable domain. In addition, the β‐hairpin peptides have been broadly explored in applications ranging from drug delivery, membrane protein stabilization, 3D cell culture, to microbial inhibition, demonstrating the potential applications of peptide tectonics in the development of biomaterials and drugs.^62^

### 3.3 Coiled‐Coil Peptide Tectons

**Figure 14:**
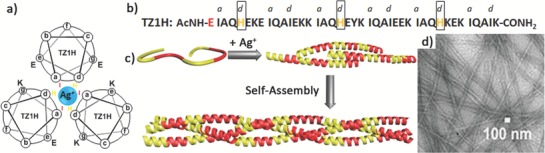
a) Helical wheel representation of peptide **TZ1H** trimeric helices coordinating with silver and b) the sequence of peptide **TZ1H**. c) Schematic illustration of the self‐assembly of peptide **TZ1H** above the p*K*_a_ of histidine residues triggered by metal coordination: a conformational transition from random coil to helix triggered by metal coordination led to formation of trimeric coiled coils that self‐assembled into nanofibers. d) TEM image of resulting nanofibers formed by peptide **TZ1H** and Ag^+^ in an equimolar ratio. Reproduced with permission.^63^ Copyright 2008, American Chemical Society.

Inspired by the pH‐sensitive self‐assembly of a derivative of isoleucine zipper GCN4‐pII peptides, Conticello and coworkers utilized a metal binding approach to switch the conformational transition of peptides and control their self‐assembly (**Figure** 14).^63^ In their initial design, they synthesized an α‐helical peptide (**TZ1H**) consisting of six heptad repeats with three histidine residues at the *d*‐position of the first, third, and fifth heptad repeats (Figure 14a).[qv: 63a] At pH values above the p*K*_a_ of histidine residues, peptide **TZ1H** was axially aligned due to the stagger of two heptad repeats from two adjacent strands, which was potentially stabilized by the complementary electrostatic interactions between the oppositely charged *e*‐ and *g*‐residues. Silver ion was selected to match the hypothetical trigonal planar geometry for histidine residues in triple stranded coiled coils. Addition of silver ions to the peptide solution at a pH value below the p*K*_a_ of histidine residues led to the conformational transition from random coils to α‐helices and the formation of triple stranded coiled coils. Thus, each peptide **TZ1H** could donate one His ligand in three independent trigonal Ag–His_3_ complexes, leading to the opportunity for the laterally staggered heptad repeats that dictate the linear assembly of coiled coils.

## 4 Cyclic Peptide Tectons

From the thermodynamic aspect for self‐assembly, restriction of the conformational space of peptide domains allows for reduction of their conformational entropy during self‐assembly, thus facilitating design of peptide tectons with predictable interacting interfaces. Cyclization of peptide domains by covalent connections is a versatile strategy to restrict their conformational space. In this context, both domains adopting a stable conformation and those undergoing a conformational transition during self‐assembly have been utilized to create cyclic peptide tectons. Regardless of the conformational stability of incorporated domains, covalent constraint of cyclic peptides confines the orientation of the functional groups within peptide backbones, thus allowing for prediction of the interacting interfaces and thereby dictating the programmable self‐assembly into well‐defined nanostructures.^64^ This section highlights several classes of cyclic peptide tectons with evidently complementary interacting interfaces and elucidates the underlying mechanism for their self‐assembly into well‐defined nanostructures.

### 4.1 Cyclic Coiled Coils

**Figure 15:**
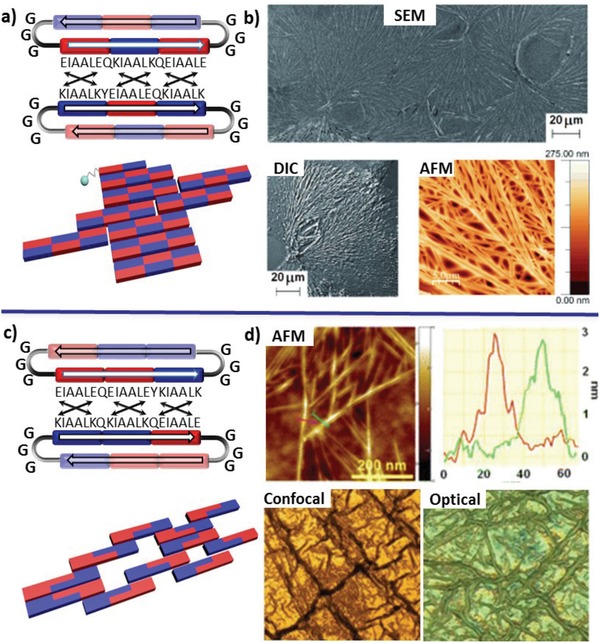
Self‐assembly of bifaceted cyclopeptides consisting of two domains with a 1 + 1 + 1 or 2 + 1 pattern for the heptads. Top: a) Schematic representation of cyclopeptide **1** containing the heptads connected in 1 + 1 + 1 symmetric model by triglycyl linkers in an antiparallel direction and the arbitrary assembly of the tectons promoted by parallel coiled‐coil heterodimers formed by the charge‐complementary domains from neighboring cyclopeptides. b) SEM, differential interference contrast, and AFM images of the nanostructures formed by cyclopeptide **1**. Reproduced with permission.^65^ Copyright 2012, Wiley‐VCH. Bottom: c) Schematic representation of cyclopeptide **2** containing the heptads connected in 2 + 1 asymmetric model within the two domains and simplified representation of the tecton assembly with one‐, two‐, and three‐heptad complementary overlaps. d) AFM, confocal, and optical microscopy images of the networks formed by cyclopeptide **2**. Blue and red boxes represent cationic and anionic heptads, respectively, while arrows denote electrostatic interactions between lysine and glutamate residues. Reproduced with permission.^65^ Copyright 2014, American Chemical Society.

In contrast to flexibly connecting coiled‐coil tectons, creation of cyclic coiled coils suffers from the synthetic challenges. Therefore, reports focusing on cyclic coiled‐coil tectons remain scarcer. One remarkable example was reported by Ryadnov and coworkers, involving cyclic helical peptides with complementary charge distribution connected by two flexible triglycyl linkers and orientated in an antiparallel direction (**Figure** 15).[qv: 50b,65] The resulting anisotropic bifaceted peptide tectons prefer to dimerize between distinct cyclic peptides, rather than intramolecular association, due to the parallel orientation for coiled coils. Within the cyclic structures, positively and negatively charged heptad repeat units were sequenced into the helical domains in a complementary fashion to each other in one cyclic peptide, including either pairing one +/−/+ domain with a −/+/− domain (cyclopeptide **1**, Figure 15a)[qv: 65a] or connecting a +/+/− domain with a −/−/+ domain (cyclopeptide **2**, Figure 15c).[qv: 65b] As a result, formation of coiled coils involving either three (and two) heptad repeats or only one heptad of the helical domains from neighboring cyclic peptides promoted lateral or longitudinal self‐assembly of cyclic peptides, eventually leading to hyperbranched fibrillar networks mimicking native extracellular matrices.

### 4.2 d,l‐α‐Cyclic Peptide Tectons

**Figure 16:**
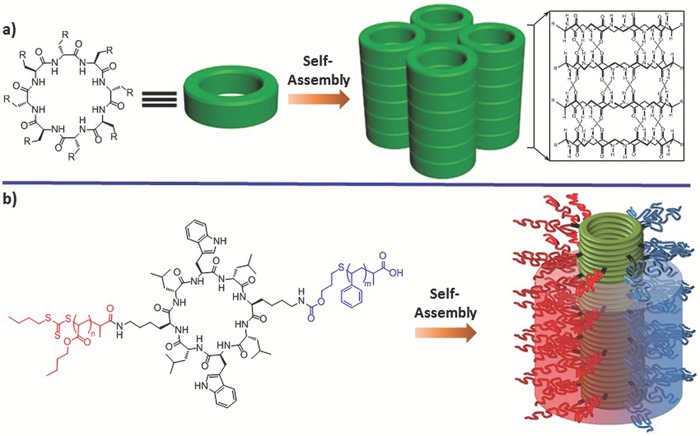
Cyclic peptide structures with alternating d‐ and l‐amino acids adopting flat ring‐shaped conformation. a) Cyclic peptides assembled into nanotubes composed of ordered parallel arrays of individual monomers predominantly based on H‐bonds. Reproduced with permission.^69^ Copyright 1996, American Chemical Society. b) Cyclic peptides served as the scaffolds for phase‐separated polymers, leading to peptide–polymer conjugates with two corona configurations, which assembled into Janus nanotubes with “demixed” corona. Reproduced with permission.^73^ Copyright 2013, Nature Publishing Group.

d,l‐Cyclic peptides have been demonstrated as ring‐shaped building blocks for creation of nanotubes with a defined interior diameter.^66^ The structural features of resulting nanotubes from d,l‐α‐cyclic peptides have been thoroughly characterized in both solution and solid crystals.^67^ The pioneering work in design and synthesis of d,l‐α‐cyclic peptides has been reported by the Ghadiri laboratory, in which the primary principle for design of cyclic peptides was alternating connection of an even number of d‐ and l‐α‐amino acid residues (**Figure** 16a).^68^ Based on the intermolecular β‐sheet‐like hydrogen bonds, self‐assembly of individual cyclic peptides stacking along the longitudinal direction leads to formation of nanotubes with an inner diameter that is associated with the size of cyclic peptides.^69^ Inspired by cyclic d,l‐α‐peptides, many cyclized sequences composed of β^3^‐amino acids or alternating α,γ‐ or 3α,γ‐amino acids, among others, have also been designed to create cyclic peptides with an analogous orientation for the side chains compared to cyclic d,l‐α‐peptides.^66^ These cyclic peptides exhibited the propensity to self‐assemble into nanotubes based on the similar principles for formation of cyclic d,l‐α‐peptide nanotubes. In particular, containing a *cis*‐3‐aminocycloalkanecarboxylic acid within cyclic α,γ‐peptides allowed for chemical modification of the inner cavity of resulting nanotubes, thus expanding the structural diversity of peptide nanotubes.^70^ Hence, cyclic peptide assembled nanotubes or the counterparts with exterior or interior functional groups have been broadly utilized as biomaterials, including transmembrane ion channels, protein modulators, drug delivery vehicles, antibacterial agents, and biosensors, among others.[qv: 64a,71]

In cyclic β‐sheet‐like peptides, selective association of cyclic peptides could be further controlled by rational incorporation of molecular or macromolecular side chains at the ring backbone. In this context, Lee and coworkers have incorporated alternating hydrophilic and hydrophobic side chains into six‐residue cyclic α‐peptide rings, leading to cyclic peptide facial amphiphiles.^72^ The facial amphiphiles utilized cyclic peptides as the scaffolds to selectively localize the hydrophilic and hydrophobic chains toward opposite face. Despite the loss of flat secondary structures of cyclic peptides, the facial amphiphiles formed discrete nanocapsules based on the hydrophobic collapse of fluorinated side chains. Based on a two‐step convergent approach, the Jolliffe and Perrier groups have orthogonally grafted two distinct polymers that are known to undergo microphase separation to eight alternating d,l‐α‐cyclic peptides, yielding asymmetric peptide–polymer conjugates (Figure 16b).^73^ Spontaneous self‐assembly of the resulting conjugates driven by hydrogen‐bonding interactions and phase separation led to fabrication of Janus nanotubes in solution and in bulk, thus potentially opening up a new versatile strategy in creation of artificial transmembrane channels.

### 4.3 Lanreotide Peptides

**Figure 17:**
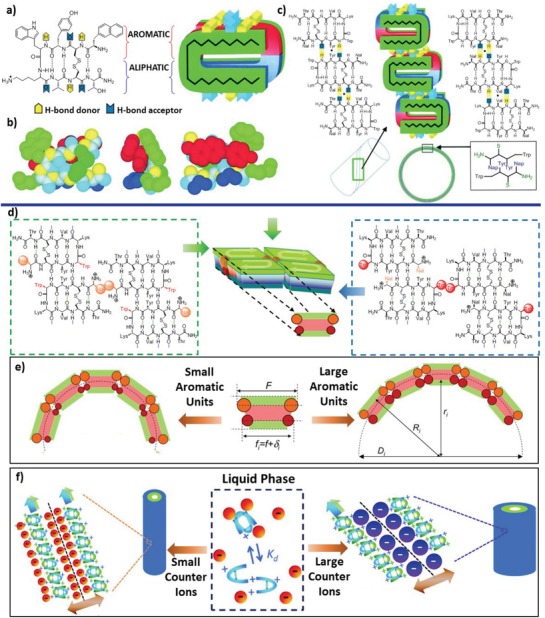
Formation of nanotubes based on hierarchical self‐assembly of lanreotide peptides. Top: Schematic representation of the mechanism of self‐assembly of lanreotide peptides. a) Chemical structures of lanreotide adopting a β‐hairpin planar conformation stabilized by the disulfide bridge, the turn, and the intramolecular H‐bonds. b) Space‐filling models of lanreotide peptides with hydrophobic and hydrophilic faces. c) In the resulting nanotubes, two different β‐sheet strands superimposed with their C_2_ twofold axes, leading to the segregation of the aromatic and aliphatic moieties on each strand of β‐hairpins. The segregation of aromatic residues (red) from aliphatic residues (blue) and from hydrophilic region (green) is highlighted. Reproduced with permission.^75^ Copyright 2003, National Academy of Sciences. Bottom: Two strategies for modulating the diameter of the nanotubes formed by lanreotide peptides. d) The stacking models of lanreotide peptides within the two layers of nanotubes. Based on the stacking models, the diameter of nanotubes can be tuned based on either e) changing the geometrical size of the aromatic unit at the position for d‐Trp residue (Reproduced with permission.^77^ Copyright 2011, National Academy of Sciences) or f) incorporation of counterions for the free amine groups with lanreotide backbones (Reproduced with permission.^77^ Copyright 2012, American Chemical Society).

Cyclic peptide tectons have also been reported by Artzner and colleagues focusing on lanreotide, which is a cyclic octapeptide synthesized as a growth hormone inhibitor into well‐defined nanotubes (**Figure** 17).^74^ At the molecular level, lanreotide, consisting of a sequence of NH_2_–(d)Naph–Cys–Tyr–(d)Trp–Lys–Val–Cys–Thr–CONH_2_, adopted a β‐hairpin conformation predominantly stabilized by the covalent constraint arising from a disulfide bond between the Cys2 and Cys7 residues, in addition to the intramolecular hydrogen bonds. The folding conformation led to localization of the hydrophobic backbone and the hydrophilic disulfide bond at opposite faces of β‐hairpin, thus giving rise to the amphiphilic nature for lanreotide (Figure 17a).^75^ Incorporation of the d‐tryptophan residue within the peptide backbone resulted in segregation of the aromatic and aliphatic moieties on each strand of β‐hairpins. The π–π stacking interactions among the aromatic segregations and the H‐bonds among intermolecular strands governed their programmable self‐assembly into nanotubes organized in a hexagonal lattice (Figure 17c).^75^ The wall of the nanotubes was formed by two superimposed layers of lanreotide peptides, where the hydrophilic residues are shielded by the hydrophilic moieties from both inner and outer β‐sheet fibers. While lanreotide peptides within one layer of the walls were aligned in a 2D monoclinic lattice and antiparallel with an identical shifting distance, the two β‐sheets of one filament were superimposed in an oppositely alternative order for the hydrogen‐bonding interfaces among β‐strands. This organizing pattern not only allows for the segregation of aromatic and aliphatic moieties, but also leads to different curvature radii for the inner and outer layers of the nanotube walls. The pathway for the self‐assembly of lanreotide peptides has been investigated to gain insight into the underlying mechanism for formation of well‐defined nanotubes.^76^ Experimental results elucidated that the self‐assembly of lanreotide proceeded through three successive stages as evidenced from formation of three distinct intermediates, i.e., peptide dimers, open ribbons, and helical ribbons. The structural features of the nanotubes are strongly associated with the size of the intermediates. For instance, the intrinsic curvature of the open ribbons and the dimerization of the peptides guide the thickness and diameter of the nanotubes, respectively.

Elucidation of the relationship between the structural features of lanreotide and its self‐assembly allows Artzner and coworkers to predict and experimentally control the size of nanotubes.^77^ This has been implemented by either mutation of d‐Trp residue (Figure 17e)[qv: 77b] or incorporation of counterions (Figure 17f)[qv: 77c] as additional structural components into the nanotube walls. The basis for the former strategy lies in the effect of the aromatic close contacts involving d‐Trp residues on the curvature radius of the inner layer of the walls (Figure 17d). Hence, changing the d‐Trp residue to other counterparts with aromatic moieties having different geometrical sizes led to the formation of monodisperse nanotubes with a diameter ranging from 9.5 to 35 nm. In the latter strategy, anionic ions have been incorporated into the nanotube walls via associating with the charged amine groups of lanreotide. Analogous to the aromatic close contacts, the counterions of the free amine groups within lanreotide backbones are likely localized within the inner layer of the walls, thus precisely regulating the diameter of the nanotubes based on the size of the anionic ions. The counterion strategy has also been utilized to create peptide nanotubes with multiple walls by replacing the monovalent to bivalent counterions.^78^ Balancing the electrostatic interaction between peptides and divalent counterions with the mechanical force arising from the stiffness of the walls led to formation of double‐walled peptide nanotubes. The resulting peptide nanotubes can serve as the templates for formation of inorganic nanostructures by mimicking biomineralization.^79^

### 4.4 Cyclic Conformational Heteropeptides

**Figure 18:**
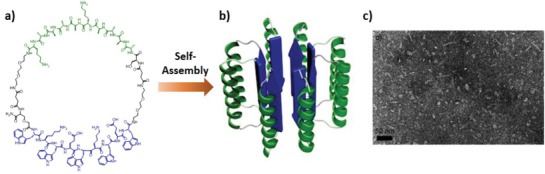
a) Macrocyclic peptides consisting of α‐helical and β‐sheet domains connected by hydrophilic flexible linkers. b) Due to the selective association among β‐sheet domains, the macrocyclic peptides assembled into spherical objects surrounded by α‐helices. c) TEM image of resulting spherical nanostructures. Reproduced with permission.^80^ Copyright 2009, Wiley‐VCH.

Alternative to the cyclization of domains with identical conformations, Lee and coworkers reported macrocyclic peptides composed of α‐helical and β‐sheet domains connected by two flexible hydrophilic oligoethylene glycol–based linkages, leading to a cyclic conformational heteropeptides (**Figure** 18).^80^ Within the macrocyclic peptides, while lysine residues in α‐helical –(KAAAA)_2_– domain render the peptide water soluble, the sequence of –(WKWE)_2_W– has a strong propensity to promote β‐sheet hydrogen‐bonding interactions.^81^ The α‐helical conformation was stabilized by the tethered β‐sheet segments within the macrocyclic peptides as characterized by circular dichroism spectroscopy. Due to the larger geometrical size of the α‐helical segment compared to the β‐sheet segments, the authors proposed that the β‐sheet segments within macrocyclic peptides underwent selective association, whereas the helices packed side by side on a sheet and rotated with respect to each other, thus leading to the formation of spherical objects analogous to α/β barrel structures. The resulting α‐helices coating spherical structures potentially enhance the efficiency for molecular recognition to substrates due to the multivalent effect.

## 5 Concluding Remarks and Future Prospects

This review introduces the concept of peptide tectonics for creation of well‐defined nanostructures driven by the selective association arising from the structural complementarity of peptide tectons. Precise prediction of the organizing pattern and interacting interface of tectons and rational incorporation of complementary units are crucial for the design of peptide tectons. Hence, peptide tectons were categorized based on their conformational entropy, which is strongly associated with the conformational stability of incorporated domains and their conformational space. The strategies for incorporation of structural complementary units to promote selective interactions among domains were highlighted to elucidate the underlying mechanism of the programmable self‐assembly in peptide tectonics.

Peptide tectonics not only provides an alternative viewpoint to understand the current status of the field of peptide self‐assembly, but also potentially facilitates the development of creation of well‐defined nanostructures. Based on the criteria for the design of peptide tectons, complementary units for robust selective association were crucial for guiding the selective association of peptides. Hence, incorporation of novel chemically functionalized units, including noncanonical amino acids^82^ or covalently bonding units,^83^ into peptide backbones as complementary structural features to further strengthen the interaction might facilitate the selective association among peptide tectons. Chemical functionalization of peptide tectons with moieties instead of amino acids leads to a considerable number of peptide conjugates such as peptide amphiphiles,[qv: 1g] drug amphiphiles,[qv: 5d,84] and conjugate amphiphiles,[qv: 4d,85] among others, which endow alternative approaches to guide the selective interactions involving abiotic moieties. For instance, while the alkyl chains and hydrophobic drugs undergo hydrophobic collapse in polar media, the aromatic drugs and conjugates promote π–π stacking interactions. The preferable self‐segregation of functional moieties provides additional driving forces for the selective association of tectons at specific interfaces. In addition, considering the limited examples, establishing reliable strategies for creation of restricted conformational space for domains potentially expands the categories of peptide tectons, particularly utilizing noncovalent bonding methods, which usually render resulting nanostructures stimulus‐responsive. Based on these considerations, peptide tectons with extraordinary complementary association at predictable interfaces could be created in the future.

Spatially and temporally precise association and organization of domains within proteins endow the sophisticated functions of native proteins, thus indicating that self‐assembly of peptides in a controllable manner facilitates development of functional materials. Peptide tectonics toward well‐defined and hierarchical nanostructures has been demonstrated as a versatile strategy to create biomaterials^86^ and energy‐related materials.[qv: 4d,87] Alternative to general peptide biomaterials, peptide tectonics allows for tuning the mechanical property of artificial matrices to manipulate cell adhesion, spreading, proliferation, and stem cell differentiation.^88^ Stimulus‐responsive self‐assembly of peptide tectons results in smart biomaterials in disease diagnosis and treatment, for instance, the sustained drug delivery,[qv: 5d,89] tumor microenvironment‐responsive therapeutics,^90^ and eliciting immune responses.^91^ In addition, peptide tectonics involving aromatic peptides or aromatic conjugate amphiphiles enables to produce nanostructures with orientation and alignment control, which have been employed to fabricate energy‐related materials, particularly for electron devices ranging from field‐effect transistors to piezoelectrics.^92^ These limited examples demonstrate the great potential of peptide tectonics in development of functional materials based on precise control over molecular interactions and organization.

Despite the progress made in design of peptide tectons, one of the predominant challenges of peptide tectonics lies in design and synthesis of conformation‐persistent complex tectons with reliable associations at predicable interfaces analogous to the tertiary subunits of native proteins. Thus far, coiled‐coil tectons are representative tertiary structures undergoing self‐assembly following desirable fashions.[qv: 1h] However, tertiary peptide tectons consisting of multiple domains adopting distinct conformation remain quite few.^80^ Incorporation of different secondary structures into tectons allows for selective interactions associated with conformational features and among domains, leading to precise creation of complex artificial systems. Combining the advantages of genetically encoded post‐translation in biosynthesis of long sequences^93^ with the computational simulation for structural optimization^94^ potentially leads to the breakthrough in design of complex tertiary tectons. In addition, precise control over the geometrical shape and size of resulting nanostructures via peptide tectonics remains challenging. Although many facile and versatile strategies have been developed to manipulate the morphology of nanostructures, the toolkit for modulating the growth of peptide tectons is limited. Benefitting from living supramolecular polymerization^95^ and kinetic‐trapped strategies,^96^ in addition to other approaches,^97^ precise control over geometrical sizes of nanostructures by peptide tectonics becomes possible. Another notable challenge in peptide tectonics is to create dynamic nanostructures with multiple functions adaptable to microenvironment. This would be the direction for development of the next generation of smart functional materials.^98^ On the basis of the continuously increasing knowledge gained from native proteins, it is promising for creation of well‐defined functional nanostructures resembling natural systems based on the programmable self‐assembly in peptide tectonics.

## Conflict of Interest

The authors declare no conflict of interest.
